# The Biofunctional Monomer, the Calcium Salt of 4-Methacryloxyethyl Trimellitic Acid, Promotes Odontoblast Differentiation in Three-Dimensional Culture System

**DOI:** 10.1155/ijbm/3693662

**Published:** 2025-06-03

**Authors:** Yaxin Rao, Youjing Qiu, Takashi Saito

**Affiliations:** ^1^Division of Clinical Cariology and Endodontology, Department of Oral Rehabilitation, School of Dentistry, Health Sciences University of Hokkaido, Tobetsu 061-0293, Hokkaido, Japan; ^2^Stomatological Hospital of Xiamen Medical College, Xiamen Key Laboratory of Stomatological Disease Diagnosis and Treatment, Xiamen 361008, China

**Keywords:** 3D culture, biofunctional monomer, calcium salt of 4-methacryloxyethyl trimellitic acid, dentin regeneration, mineralization, odontoblast differentiation, type I collagen

## Abstract

This study evaluated the effects of the biofunctional monomer CMET on the proliferation, differentiation, and mineralization of MDPC-23, odontoblast-like cells in a three-dimensional (3D) culture system using type I collagen. CMET (0.3%, w/v) facilitated the early adhesion and spreading of the cells in type I collagen gels. It significantly promoted cell proliferation in 0.2% and 0.3% concentrations. ALP activity also increased in the 0.3% CMET group. The 0.3% CMET group markedly enhanced odontogenic differentiation by upregulating mRNA of odontogenic differentiation markers such as DSPP and DSP-1. Mineral nodule formation in MDPC-23 cells grown in the 0.3% CMET group was markedly increased compared to that in the control group. After treating the cells with the three MAPK inhibitors, the ability of CMET to stimulate ALP activity in MDPC-23 cells was totally suppressed to control levels by the p38 inhibitor, SB202190. The enhancement of mineralization of MDPC-23 by CMET was partially impeded by SB202190. The results demonstrated that the biofunctional monomer CMET induced proliferation, differentiation, and mineralization of odontoblast-like cells in a 3D culture system using type I collagen gel at a concentration of 0.3%. Thus, combining CMET and type I collagen gel as a scaffold does not exhibit apparent cytotoxicity and is suggested to have immense potential for dentin regeneration.

## 1. Introduction

Vital pulp therapy (VPT), particularly direct pulp capping, aims to preserve the vitality and function of teeth with damaged or infected pulps. This therapy is typically used when the pulp is compromised owing to trauma, deep cavities, or other injuries. The primary goals of VPT are to alleviate pain, prevent infection, promote healing, and continue tooth development, especially in young and developing teeth [1]. Moreover, VPT is a preferred alternative to tooth extraction because maintaining a natural tooth is often the best option for overall oral health and function. The success of such treatments depends on several factors, with biocompatibility being a key factor [2]. Biocompatible materials used in endodontics should not cause inflammation, necrosis, or other adverse reactions in the pulpal tissue. These materials should also have properties that promote the formation of a dentin bridge or layer of reparative dentin, which helps protect the pulp and prevent infection. Commonly used biocompatible materials in endodontics include calcium hydroxide (CH), mineral trioxide aggregates (MTA), and various bioceramic materials. These materials exhibit good biocompatibility and are widely used in VPT. Additionally, CH is a standard pulp-capping material with a long-term track record in clinical application [3, 4]. However, shortcomings, such as mechanical instability, poor sealing ability [3], and poor quality of the resulting dentin bridge [5], have been reported with CH.

MTA is a bioactive endodontic cement composed mainly of calcium and silicate elements [6] and is used as an alternative to CH because of its excellent sealing properties and biocompatibility [7]. However, it indicates some shortcomings, such as difficult operation, long setting time, high cost, and tooth discoloration [7]. In recent decades, resin materials have been used in endodontic treatment and proposed as potential pulp-capping agents [8]. Pulp-capping agents containing resinous materials have several advantages, the most prominent of which are excellent adhesion and effective sealing against microleakage. However, compared with CH-based cement, inferior results have been reported when using resin materials for pulp capping due to their potential toxicity [9]. Therefore, materials with good sealing properties and biocompatibility should be developed.

**Figure 1:**
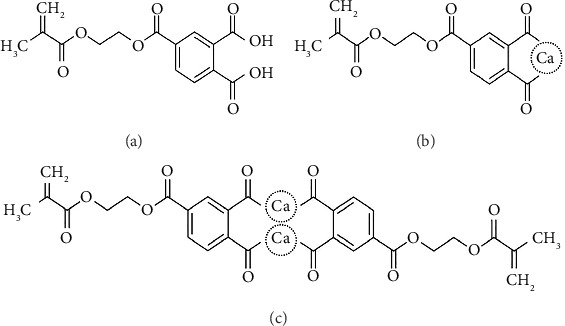
Chemical structure of 4-methacryloxyethyl trimellitic acid (a) monomer (b) and dimer (c) of calcium salt of 4-methacryloxyethyl trimellitic acid (CMET).

A previous study demonstrated that 4-methacryloxyethyl trimellitic acid (4-MET) (Figure 1(a)) could enhance the resin bonding to the enamel and dentin because of the potential of the hydrophilic group to form a chemical bond with the calcium of hydroxyapatite [10].

Our research group recently developed (Figures 1(b) and 1(c)) a calcium salt of 4-MET, called the CMET, as a biofunctional monomer. CMET was produced by replacing the hydrogen ions in the two carboxyl groups with the calcium ion(s) in 4-MET for neutralization. CMET exists both as a monomer and a dimer [11]. Owing to the additional function of 4-MET, CMET has some benefits, such as inducing the remineralization in decalcified dentin, enhancing the mechanical properties of resin materials [11], and inhibiting the formation of *Streptococcus mutans* biofilms [12]. Additionally, CMET exhibited low cytotoxicity, high differentiation-inducing ability, and mineralization in odontoblast-like cells [13]. Owing to the abovementioned advantages, CMET is expected to be used as a potential pulp-capping agent.

Collagen, specifically type I collagen (COL-I), is the primary component of the extracellular matrix in animal connective tissues. COL-1 is abundant, biocompatible, and bioabsorbable, which makes it an ideal biological scaffold. It can self-assemble, providing a structural framework for cell attachment and interaction. Using COL-1 as a scaffold in tissue engineering offers excellent potential for tissue regeneration and repair [14]. The molecular structure of collagen consists of a right-handed triple helix region formed by two α1 and one α2 chains and a telopeptide region (nonhelical region) at the N-terminus and C-terminus. The triple-helix region is genetically conserved across species; it exhibits low antigenicity in vivo, whereas the telopeptide region exhibits high antigenicity. Atelocollagen is obtained by cleavage with a protease and subsequent removal of the telopeptide region. Atelocollagen has the same properties as collagen but low antigenicity [15]. Therefore, COL-I is a possible carrier of CMET when used for VPT and a scaffold for odontoblast proliferation and differentiation.

Cell culture simplifies biological systems, reduces time and resources, and is more ethical than in vivo experiments. Despite these advantages, conventional in vitro cell cultures cannot fully mimic the complex environment of the body or respond to intricate stimuli [16]. The superiority of three-dimensional (3D) cell culture over two-dimensional (2D) cell culture has been increasingly recognized in recent years, owing to its ability to emulate complex physiological cues better and support long-term cell viability, making it an attractive option for various biomedical applications [17]. A previous study identified that COL-I maintains its nonfibrous form under acidic pH [18]. Coating the surface of cell culture dishes with the nonfibrous COL-1 promotes cell adhesion and proliferation. At a physiological pH and temperature, nonfibrous COL-I molecules reassemble into fibrils, forming gels with a 3D structure [19]. Therefore, COL-I can be a scaffold for 3D cell culture under physiological conditions.

Previously, we studied the role of CMET in a 2D environment in vitro. To emulate the complex in vivo environment, this study intended to evaluate the effects of the biofunctional monomer CMET on odontoblast proliferation, differentiation, and mineralization in a COL-I 3D culture system used for future clinical applications of CMET.

## 2. Materials and Methods

### 2.1. CMET Treatment

The material used in these experiments was CMET in COL-I (0.2%, v/v) neutral solution (Bovine skin atelocollagen, DME-02H, Lot. no.354029, KOKEN). Regarding the combination of 0.1%–0.6% CMET in DME-02H solution, 20 mL of DME-02H was first stored in a refrigerator for a few days and then thawed. CMET was crushed using an agate mortar and pestle. For the preparation of 0.1% CMET in DME-02H, the powder of 20 mg CMET was mixed with 20 g of DME-02H in 30-mL clear brown glass sample bottles. The mixture was stirred using a magnetic stirrer in an ice bath for 1 h, ensuring that uniform dissolution was confirmed to dissolve. The mixture was then stored in a freezer until further use. The other concentrations of CMET in DME-02H were prepared similarly. The participants were divided into two groups. In Experiment 1, the CMET was diluted to 0%, 0.1%, 0.2%, 0.3%, 0.4%, 0.5%, and 0.6% (w/v) for addition into the medium with COL-I neutral solution. Based on Experiment 1, 0.3% (w/v) CMET in a COL-I neutral solution was selected as the optimal concentration to explore the mechanism of CMET in experiment two. In both experiments, 0.0% (w/v) gel-CMET was used as a control.

### 2.2. Cell Culture

Immortalized mouse dental papilla cell-23 (MDPC-23) cells, an odontoblast-like cell line, were used in this study. The cell suspensions were mixed with different concentrations of CMET and inoculated onto nontissue culture-treated plates. On Day 1, the cover medium was added when the collagen became a gel. The cells were cultured in Dulbecco's modified Eagle's medium (DMEM, Sigma-Aldrich, St. Louis, MO, USA) supplemented with 5% fetal bovine serum (FBS, Gibco, Grand Island, NY, USA). The cover medium was changed every alternate day. All cells were cultured in a 37 C humidified incubator with an atmosphere of 5% carbon dioxide and 95% air. Passage 27 cells were used for subsequent experiments.

### 2.3. Cell Proliferation Assay

MDPC-23 cells (5 × 10^3^ cells/well) were seeded in 96-well nontissue culture-treated polystyrene plates (Corning, NY, USA). Cell viability was assessed by Cell Counting Kit-8 (CCK-8) assay on Day 4. The CCK-8 (Dojindo, Rockville, MD, USA) reagent was added to each well (10 μL/well) and incubated for 1 h and 30 min. The absorbance of the lysates was measured at 450 nm using a microplate reader (Bio-Rad, Hercules, CA, USA).

### 2.4. Cell Morphology and Cell Number Observation

MDPC-23 cells (4 × 10^4^ cells/well) were seeded in 12-well polystyrene plates. In Experiment 1, cells were visualized under a phase-contrast microscope (Olympus, Shinjuku, Tokyo, Japan) from Day 1 to Day 6. This study compared the cell morphology and number of different concentrations of CMET (0.0%, 0.1%, 0.2%, 0.3%, 0.4%, 0.5%, and 0.6%, w/v) in COL-I.

### 2.5. Alkaline Phosphatase (ALP) Activity Assay

The MDPC-23 cells (4 × 10^4^ cells/well) were cultured in 12-well plates. Mineralization-inducing reagents, 10 mmol/L glycerol-2-phosphate disodium salt n-hydrate (β-GP, Wako, Osaka, Japan), 50 μg/mL L-ascorbic acid phosphate magnesium salt n-hydrate (AA, Wako), and 100 nM/mL dexamethasone (Dex, Sigma-Aldrich) were added to DMEM (5% FBS) on Day 5 when the cells reached confluence. Mineralization analyses and quantitative real-time reverse transcription polymerase chain reaction (RT-PCR) were performed according to the protocols described above. Mineralization-inducing capacity was evaluated by ALP activity assay. On Day 6, cells within the gel were transferred to a 1.5-mL tube and digested with type I collagenase (Worthington, Columbus, OH, USA) diluted in Hank's balanced salt solution (HBSS, Gibco, Grand Island, NY, USA) at 37°C for over 1 h until the gel was digested. Cells were collected by centrifugation (12, 000 rpm, 5 min, 37°C) and lysed with 0.1% Triton X-100 (Sigma-Aldrich) in distilled water. The lysates were sonicated on ice for 10 min and then centrifuged at 12, 000 g at 4 C for 15 min. According to the manufacturer's instructions, the supernatant was extracted for ALP activity assay using LabAssay ALP activity kit (Wako), and protein quantification using a Pierce BCA protein assay kit (Thermo Fisher Scientific, Waltham, MA, USA). The absorbance was measured at 405 and 570 nm.

### 2.6. Quantitative Real-Time RT-PCR

On Day 6, cells in the gel were transferred to a 1.5-mL tube digested with type I collagenase diluted in HBSS at 37°C for over 1 h until the gel was digested. Cells were collected by centrifugation (12, 000 rpm, 5 min, 37 C), and total ribonucleic acid (RNA) was extracted using TRIzol reagent (0.5 mL/well, Invitrogen, Carlsbad, CA, USA). After incubating for 5 min, 0.1 mL of chloroform (Nacalai Tesque, Kyoto, Japan) was added to each tube. The mixture was vigorously shaken for 15 s and incubated at room temperature for 2 min. The samples were centrifuged at 12, 000 rpm for 15 min at 4°C. The aqueous phase was transferred to a new tube, mixed with 0.25 mL 2-propanol (Nacalai Tesque), and incubated at room temperature for 10 min. The samples were centrifuged at 12, 000 rpm for 10 min at 4 C.

The isolated RNA was pelleted, washed with 75% ethanol, and resuspended in nuclease-free water. The RNA concentration of each sample was measured using a NanoDrop ND-1000 (Thermo Fisher Scientific, Rockford, IL, USA). Additionally, according to the manufacturer's instructions, 1 μg of isolated RNA was reverse-transcribed into complementary DNA (cDNA) using M-MLV reverse transcriptase in a 20-μL reaction system. The resulting cDNA was subjected to quantitative RT-PCR.

To evaluate cell differentiation, odontogenic gene messenger RNA (mRNA) expression was quantified using the primers by LightCycler Nano (Roche, Basel, Switzerland) according to the manufacturer's instructions. Target gene expression was normalized to the housekeeping gene rat β-actin. The comparative 2^−△△CT^ method was used to calculate relative gene expression. Primer sequences and reaction conditions used for real-time RT-PCR are listed in Table 1.

### 2.7. Alizarin Red S (ARS) Staining

ARS staining was performed on Day 7 to detect calcific deposits in the cells. The cells were washed twice with 1 × phosphate-buffered saline (PBS, Gibco), fixed with 10% formalin neutral buffer solution (Thermo Fisher Scientific) for 20 min, and washed again with distilled water (dH_2_O) once. ARS (1% m/v, pH 4.1) was carefully added and incubated at 37°C for 10 min. To avoid excessive staining and removal of nonspecific background staining, the monolayer was washed several times with dH_2_O until the water appeared clear. The calcific deposition was visualized using phase-contrast microscopy. To quantify the calcific staining intensity, ARS staining images were processed using ImageJ (National Institutes of Health, Bethesda, MD, USA) to extract data.

### 2.8. Selective Inhibition of MAPK

To investigate the effects of the inhibitors on cell differentiation and mineralization, the cell suspension was mixed with 0.3% (w/v) gel-CMET and then inoculated at an initial density of 4 × 10^4^ cells/well in 12-well plates either treated or nontreated. On Day 1, cells were covered with 20 μM of one of three mitogen-activated protein kinase (MAPK) pathway inhibitors, SP600125 (Cell Signaling Technology, Danvers, MA, USA) for c-Jun N-terminal kinase (JNK), SB202190 (Cell Signaling Technology) for p38, and PD98059 (Cell Signaling Technology) for extracellular signal-regulated kinase (ERK) dissolved in dimethyl sulfoxide in DMEM supplemented with 5% FBS. Then, 10 mM β-GP and 50 μg/mL AA were added to DMEM (5% FBS) with inhibitors on Day 5 when the cells reached confluence. The control group was composed of 0.0% (w/v) CMET gel. The following analyses of ALP activity and mineralization were performed according to the protocols described above.

### 2.9. Statistical Analysis

All experiments were carried out in triplicate, and the results are expressed as the mean ± standard deviation. Data were subjected to a one-way analysis of variance with post hoc Tukey's honestly significant difference test, and *p* < 0.01 was considered statistically significant.

## 3. Results

### 3.1. Cell Proliferation

**Figure 2:**
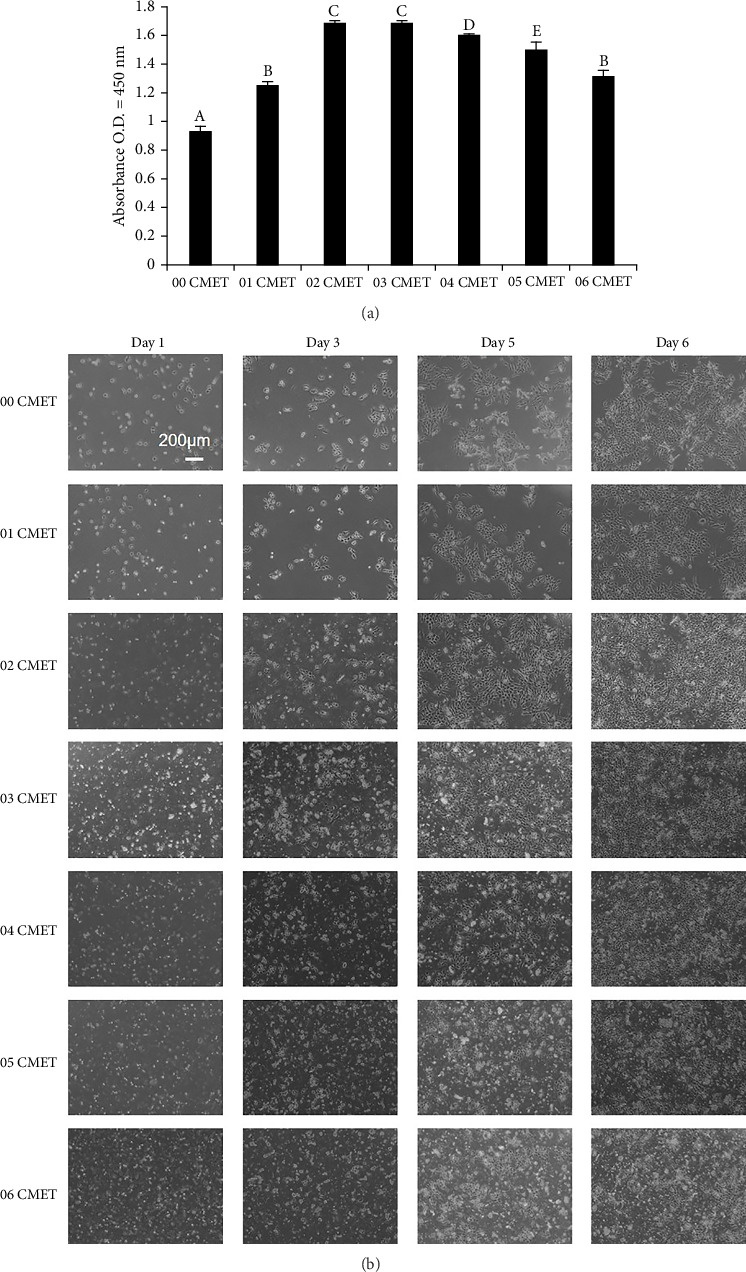
(a) Effects of calcium salt of 4-methacryloxyethyl trimellitic acid (CMET) on the proliferation of mouse dental papilla cell-23 (MDPC-23) cells (*p*=27). Cell proliferation is detected using the cell counting kit-8 assay on Day 4. Different uppercase letters indicate a significant difference (*p* < 0.01). (b) Cell morphology and number observation of MDPC-23 cells. Cell morphologies are visualized using phase-contrast microscopy from Day 1 to Day 6. The scale bar in the control group image of Day 1 applies to all panels (bar = 200 μM).

Cell proliferation was evaluated using the CCK-8 assay. CMET promoted MDPC-23 cell proliferation in all experimental groups in a dose-dependent manner. In comparing different concentrations of gel-CMET, the 0.2% (w/v) and 0.3% (w/v) gel-CMET groups reached the maximum among all groups (*p* < 0.01) (Figure 2(a)).

### 3.2. Cell Morphology and Number Observation

The cell morphology was visualized using phase-contrast microscopy from Day 1 to Day 6 (Figure 2(b)). In the 3D environment of COL-I formation, cells in all groups successfully attached, spread, and adopted a spindled shape. On Day 1, the cells in all groups were round and spot-like. On Day 2, the cells started to flatten in the 0.0%–0.3% (w/v) gel-CMET groups, whereas those in the 0.4%–0.6% (w/v) CMET groups retained a round shape. The number of cells in the 0.3% (w/v) gel-CMET group was significantly higher than that in the 0.0% (w/v) gel-CMET group. By Day 3, cells in the 0.0%–0.3% (w/v) gel-CMET groups had elongated, with a significant increase in cell number, while cells in the 0.4%–0.6% (w/v) gel-CMET groups remained round. On Day 4, the cell morphology in the 0.4%–0.6% (w/v) gel-CMET groups was similar to the morphologies in the other groups. On Day 5, the differences between the surfaces were significantly evident. The cells in the 0.3% (w/v) gel-CMET group reached confluence. By Day 6, the cells in all groups had elongated and reached confluence. According to cell morphology observations, 0.3% (w/v) CMET facilitated early adhesion and spreading of MDPC-23 cells in COL-I gels.

### 3.3. ALP Activity

**Figure 3:**
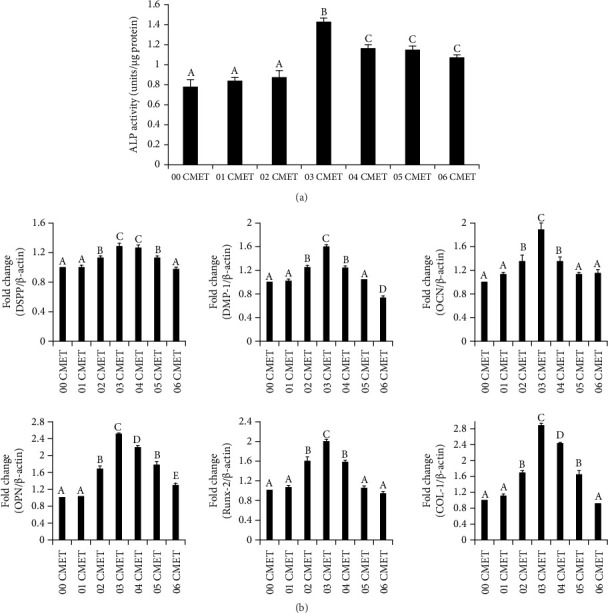
(a) Relative alkaline phosphatase (ALPase) activity on Day 6. The samples are analyzed using a LabAssay alkaline phosphatase (ALP) kit (Wako), and total protein is quantified with a bicinchoninic acid protein assay kit (Pierce). ALP production is normalized to the total protein amount. Different uppercase letters indicate a significant difference (*p* < 0.01). (b) The messenger ribonucleic acid expression of odontogenic differentiation markers (dentin sialophosphoprotein [DSPP], dentin matrix acidic phosphoprotein 1 [DMP-1], osteocalcin [OCN], osteopontin [OPN], Runt-related transcription factor 2 [Runx-2], and type I collagen [COL-1]). Different uppercase letters indicate a significant difference (*p* < 0.01).

To detect early-stage cell differentiation toward the odontogenic lineage, an ALP activity assay was performed on Day 6. The 0.1% (w/v) and 0.2% (w/v) gel-CMET groups displayed no significant differences compared to the 0.0% (w/v) gel-CMET group. In comparing different concentrations of gel-CMET, ALP activity was significantly augmented in the 0.3% (w/v) gel-CMET group. The facilitative effect decreased with increasing concentration; however, ALP activity in the 0.4% (w/v), 0.5% (w/v), and 0.6% (w/v) gel-CMET groups was still higher than that in the 0.0% (w/v) gel-CMET group (Figure 3(a)).

### 3.4. Quantitative Real-Time RT-PCR

Quantitative real-time RT-PCR was performed to investigate the effects of gel-CMET on odontogenic differentiation. The 0.3% (w/v) gel-CMET group strongly enhanced the mRNA expression of critical odontogenesis-related markers: dentin sialophosphoprotein (DSPP), dentin and dentin matrix acidic phosphoprotein 1 (DMP-1), osteocalcin (OCN), osteopontin (OPN), runt-related transcription factor 2 (Runx-2), and COL-I in the comparison of different concentrations of gel-CMET (*p* < 0.01). The RNA expression of DMP-1 slightly decreased in the 0.6% (w/v) gel-CMET group (Figure 3(b)).

### 3.5. ARS Staining

**Figure 4:**
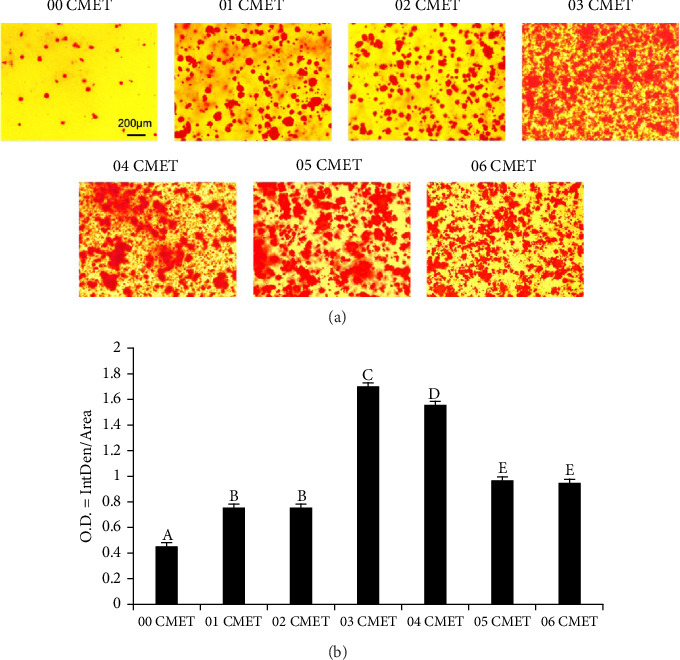
(a) Photographs of mineral nodules formed after 7 days of culture under phase-contrast microscopy. The scale bar in the control group image applies to all panels (bar = 200 μM). (b) Quantification of mineral nodules formed using ImageJ. Different uppercase letters indicate a significant difference (*p* < 0.01).

To evaluate the effects of gel-CMET on the promotion of a mature odontoblast phenotype, ARS staining was performed on Day 7. The results demonstrated that gel-CMET significantly promoted calcium nodule formation in MDPC-23 cells in a concentration-dependent manner in the presence of mineralization-inducing reagents (Figure 4(a)). Images of ARS staining were processed using ImageJ to extract data (O.D. = IntDen/Area), which confirmed a significant increase in mineralization in the 0.3% (w/v) gel-CMET group relative to the control group (*p* < 0.01) (Figure 4(b)).

### 3.6. Selective Inhibition of MAPKs

**Figure 5:**
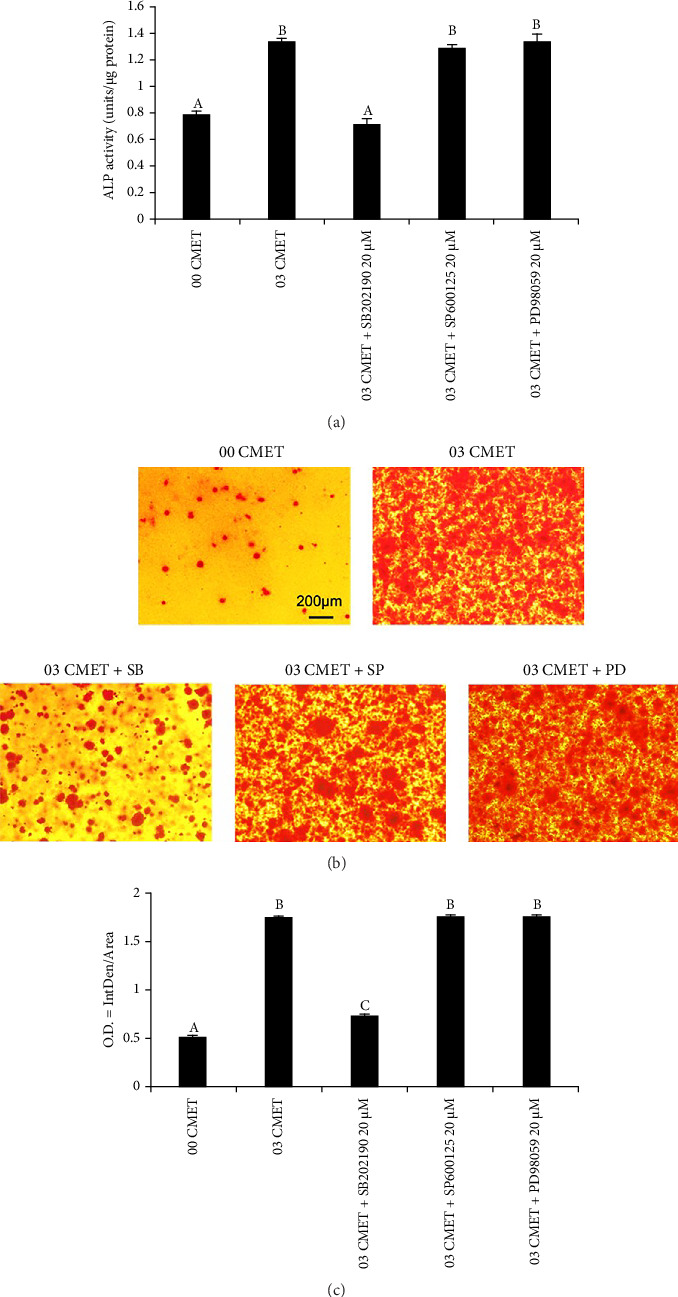
(a) Effects of inhibition of p38, c-Jun N-terminal kinase (JNK), or extracellular signal-regulated kinase (ERK) activation on alkaline phosphatase activity (SB202190 for p38, SP600125 for JNK, and PD98059 for ERK). (b) Photographs of mineral nodules formed after inhibition of p38, JNK, or ERK activation. (c) Quantification of mineral nodules formed after inhibition of p38, JNK, or ERK activation using ImageJ. Different uppercase letters indicate a significant difference (*p* < 0.01).

After challenging the cells with the three MAPK inhibitors, ALP activity, which is essential for biomineralization, was analyzed. The results demonstrated that SB202190 neutralized the positive effect of CMET on ALP activity to a level comparable with that of the control group (Figure 5(a)). In the ARS staining experiment, the enhancement of cell mineralization by CMET was partially inhibited by blocking the p38 MAPK signaling pathway with SB202190; however, it was still augmented compared to that of the control group. ImageJ quantification data also supported this finding (Figures 5(b) and 5(c)).

## 4. Discussion

As is well established, many tissues exhibit 3D spatial features. The 2D cell culture systems have limitations in studying complex biological processes, such as morphogenesis, tissue remodeling, and cancer cell migration. However, 3D culture systems provide a realistic and physiologically relevant environment for studying these processes, as they better replicate the 3D nature of tissues and organs in the human body [20]. Studies have discovered that owing to differences in cell morphology, cell surface receptor organization, and cell stage [21–24], cells cultured in 3D display different responses to drugs than those cultured in 2D.

A previous study established that keratinocyte (FEPE1L-8) proliferation was slower on the fibril form of COL-I (3D) than on the nonfibrous form (2D) [19]. Another study discovered that an on-gel culture using COL-I affected cancer cells. In the fibril form of COL-I, Akt activation and the growth of Caco-2 cells (a colon cancer cell line) are suppressed [25]. Additionally, the growth of human melanoma cells (M24met) in the fibril form is arrested at the G1/S checkpoint [26]. Moreover, markedly increased levels of reactive oxygen species (ROS) have been observed in murine 3T3-L1 preadipocytes cultured in fibril form [27]. This revealed that the fibril form of COL-I only provided an environment mimicking the body rather than facilitating cell proliferation.

According to the CCK-8 assay results, CMET not only exhibited low cytotoxicity but also promoted cell proliferation at a concentration of 0.3% (w/v). After reviewing the dental literature, the following conclusions were drawn regarding the cytotoxicity of the monomers: A previous study discovered that hydrophobic monomers have higher cytotoxicity than hydrophilic monomers, as they cause detrimental alterations in the cell membrane and induce morphological changes [28]. The 4-MET shows hydrophilic nature and 4-MET-containing resin indicates biocompatibility to dental pulp cells [29, 30]. We developed a calcium salt of 4-MET based on the concept of modifying the structure of dental material conventionally used in dental caries treatment. Our previous study identified that the solubility of CMET in water is high [13], revealing its hydrophilic nature and suggesting that its cytotoxicity is relatively low. We compared cell viability among CMET, 4-MET, and CH, and then, found that the concentration of CMET up to 20% and 4-MET up to 15% showed good viability of MDPC-23, while 5% of CH decreased it [13]. Thus, the 0.1%–0.6% range of CMET and 4-MET applied in this study does not show any harmful effect to odontoblasts. Thus, CMET and 4-MET are suggested to be biocompatible compared to CH, a conventional VPT material. The animal study showed the superiority of CMET powder as a biocompatible potent inducer of dentin regeneration and supported the results of the in vitro study [13]. Additionally, disturbances in pH homeostasis may induce apoptosis via various mechanisms, some of which are related to the control of mitochondrial ROS production, resulting in cell cycle arrest [31]. Further investigation regarding the metabolic mechanism of CMET's effects in MDPC-23 would help to prove that it is a safe and effective VTD material. Furthermore, COL-I was also present at physiological pH, which explains its low cytotoxicity in MDPC-23 cells.

Odontoblasts originate from the dental papilla and are a highly specialized cell line that forms dentin by secreting collagen and noncollagenous organic matrix components and controlling mineralization throughout its life cycle [32]. Over the past 20 years, several genes highly expressed during odontoblast differentiation have been identified. As dentin is a bone-like tissue, the extracellular matrix of the dentine is similar to the bone matrix. Therefore, although COL-I is the major organic component, the extracellular dentin matrix also contains a variety of noncollagenous proteins. For instance, DSPP is highly expressed in dentin; DMP-1 is related to hard tissue formation, especially dentin; Runx2 is critical for odontoblast differentiation; and OPN and OCN are expressed in dentin [33–37]. Our previous study demonstrated that a bioactive universal bond with CMET promotes proliferation, differentiation, and mineralization in vitro [38]. In the present study, conducted in a 3D culture environment, we observed consistent results: 0.3% (w/v) gel-CMET markedly enhanced odontogenic differentiation, as evidenced by upregulated mRNA expression of odontogenic markers such as DSPP, DMP-1, OCN, OPN, Runx2, and COL-I, along with increased ARS staining. While gene expression data strongly support the differentiation-promoting effect of CMET, protein-level validation in future studies would further substantiate our interpretation of the present findings and strengthen the scientific basis for this novel bioactive monomer.

Calcium ions play essential roles in cell adhesion, proliferation, and differentiation. As a calcium salt of 4-MET, CMET releases calcium ions [11]. Therefore, it can be speculated that activating specific signaling pathways by intermediary calcium ionotropic membrane receptors [39] releases calcium ions from CMET. In recent years, researchers have studied pathways related to the odontogenic differentiation of odontoblasts. The wnt signaling pathway promotes tissue repair and regeneration [40–43]. Previous studies have revealed that the bone morphogenetic protein/Smad signaling pathway can regulate DSPP both in vitro and in vivo and plays a crucial role in regenerating the dentin–pulp complex [34, 36, 44]. The nuclear factor kappa-light-chain-enhancer of activated B cells (NF-κB) signaling pathway is involved in a variety of biological processes, such as cell proliferation and apoptosis, inflammation, and immune response, and plays a vital role in the odontogenic differentiation of dental pulp stem cells (DPSCs) [45–47]. The MAPK pathway is a signal transduction pathway that couples intracellular responses to the binding of growth factors to cell surface receptors and is activated during odontoblast stimulation in tertiary dentinogenesis [48]. The MAPK pathway is a well-known signal transduction pathway that transmits signals from the cell surface receptors to the nucleus. This pathway is critical for various cellular processes, including growth, differentiation, and response to external stimuli, such as growth factors [49]. Studies have discovered that most signaling pathways interact with the MAPK signaling pathway. The MAPK signaling pathway is essential in promoting the odontogenic differentiation of DPSCs [50].

Therefore, in this study, the MAPK signaling pathway was hypothesized to be the mechanism underlying the in vitro effects of CMET on MDPC-23 cells. The MAPK family comprises three main subfamilies: ERK, p38 MAPK, and JNK [51]. The ERK signaling pathway is a characteristic of the MAPK signaling pathway and is involved in the regulation of cell differentiation [52]. The p38 MAPK signaling pathway regulates cytokine expression and is activated by inflammatory cytokine signals [53–55]. The JNK signaling pathway involves a stress-activated kinase associated with antiproliferative and apoptotic functions [55, 56]. A study identified that when differentiation, induction, and calcification were stimulated, the activity of the p38 MAPK and JNK pathways increased [57].

To investigate the involvement of the MAPK pathway in mediating the effects of CMET on MDPC-23 cells, three specific MAPK inhibitors were used in the present study: SB202190, SP600125, and PD98059. These inhibitors target different components of the MAPK pathway. SB202190 is a selective inhibitor of p38α and β isoforms, while SP600125 inhibits the phosphorylation of a protein called c-Jun, specifically targeting JNK function. PD98059 blocks ERK activation by binding to MAPK kinase 1 and prevents its activation using upstream protein kinases. To ensure minimal cytotoxicity, we utilized the lowest maximal working concentrations of the three MAPK inhibitors as recommended by the manufacturer.

ALP activity is an important determinant for evaluating osteoblast and odontoblast-initiated differentiation and is directly related to tertiary dentin production [58]. The present results demonstrate that the relative ALP activity of the 0.3% (w/v) CMET group was significantly higher than that of the other groups. The ability of CMET to stimulate MDPC-23 cells was reduced entirely to control levels by the p38 MAPK inhibitor SB202190, whereas the JNK inhibitor SP600125 and ERK inhibitor PD98059 exhibited no change compared with the CMET group, indicating that the upregulation of ALP activity induced by CMET was primarily mediated by the p38 MAPK signaling pathway rather than the ERK or JNK pathways. Moreover, ARS staining was used to detect calcific deposits in the cells. The results indicated that 0.3% (w/v) CMET markedly promoted the mineralization of MDPC-23 cells. The enhancement of cell mineralization ability by CMET was partially inhibited by blocking the p38 MAPK signaling pathway, whereas the JNK and ERK pathways exhibited no alteration compared to the CMET group. This suggests that the role of CMET in cell mineralization induction was not entirely dependent on the activation of the p38 MAPK signaling pathway, implying the involvement of other signaling pathways in CMET-induced MDPC-23 cell mineralization.

In summary, within the limitation of our experimental methodology, our results demonstrated that 0.3% (w/v) CMET in COL-I induced the proliferation, differentiation, and mineralization of odontoblast-like cells in vitro. As 3D cell culture can mimic the body's environment, this indicates the possibility that CMET plays a stimulatory role in vivo, suggesting the great potential of CMET in future VPT.

## 5. Conclusion

The present study's findings indicate that the bioactive monomer CMET induces proliferation, differentiation, and mineralization of odontoblast-like cells in a 3D culture system using COL-I at a 0.3% (w/v) CMET. An investigation of the effects of specific cell signaling pathway inhibitors revealed that the p38 MAPK inhibitor SB202190 was the most effective in differentiation and mineralization in odontoblast-like cells, suggesting the involvement of the p38 MAPK pathway.

Although within the limitation of our experimental methodology, combining CMET and COL-I gel as a scaffold does not exhibit cytotoxicity and is suggested to possess the great potential for dentin regeneration, making it a promising material for future VPT.

## Figures and Tables

**Table 1:** Rat primer sets used for quantitative real-time reverse transcription polymerase chain reaction (RT-PCR).

Target gene	Primer sequence	Product size (bp)
DMP-1	Forward: 5′-CGTTCCTCTGGGGGCTGTCC-3′ Backward: 5'-CCGGGATCATCGCTCTGCATC-3′	577

OCN	Forward: 5′-AGCTCAACCCCAATTGTGAC-3′ Backward: 5′-AGCTGTGCCGTCCATACTTT-3′	190

OPN	Forward: 5′-TTTCCCTGTTTCTGATGAACAGTAT-3′ Backward: 5′-CTCTGCTTATACTCCTTGGACTGCT-3′	228

DSPP	Forward: 5′-TCAATGGCGGGTGCTTTAGA-3′ Backward: 5′-TGCTCACTGCACAACATGAAGA-3′	111

Runx-2	Forward: 5′-CCACAGAGCTATTAAAGTGACAGTG-3′ Backward: 5′-AACAAACTAGGTTTAGAGTCATCAAGC-3′	87

Col1*α*1	Forward: 5′-ATCAGCGTAAAGAGGTCTGCC-3′ Backward: 5′-GTACAGGCCTTTGTGTGGGT-3′	173

β-actin	Forward: 5′-AACCCTAAGGCCAACAGTGAAAAG-3′ Backward: 5′-TCATGAGGTAGTCTGTGAGGT-3′	240

## Data Availability

The data that support the findings of this study are available from the corresponding author upon reasonable request.
